# Contacting Patients After an Emergency Department Visit to Influence their Follow-Up Care Preferences

**DOI:** 10.51894/001c.7004

**Published:** 2018-09-26

**Authors:** Matthew C. Bombard, Hannah CM Koaches, Omar J. Francis

**Affiliations:** 1 Emergency Medicine Physician Reston Hospital Center, Reston, VA; 2 PGY4 Emergency Medicine Resident, Henry Ford Macomb Hospital, Clinton Township, MI; 3 Clinical Faculty Supervisor, Emergency Medicine Physician, Henry Ford Macomb Hospital, Clinton Township, MI

**Keywords:** patient follow-up, post-discharge phone call, emergency department care

## Abstract

**CONTEXT:**

Emergency Departments (ED) have faced increasing challenges in providing quality, cost-effective patient care. In addition, healthcare administrators have sought specific techniques to improve patient perceptions of care and satisfaction as a component of Medicare reimbursement and physician contract retention. This five-month study sought to examine whether contacting patients per phone or leaving them a voicemail message after an ED visit might influence their perceptions of care and subsequent follow-up care preferences.

**METHODS:**

A sample of 95 discharged ED patients were contacted by phone and mailed surveys rating their likelihood of return directly for future ED as well as scheduling office-based visits. Patients were stratified by whether they were: a) directly spoken to over the phone, b) left a voicemail message, or c) never successfully contacted. Mailed patient surveys utilized a five-point Likert-type scale items concerning future follow-up care preferences. Sample patients were also monitored in the electronic health record to correlate self-reported intentions with whether they actually returned to the same ED for the same chief complaint within 30 days of their initial visit.

**RESULTS:**

Those patients who were directly contacted after ED discharge tended to be more likely to report they would return to the same ED, although not significantly (p = 0.060). Patients who were left a voicemail message were not more likely to return to the ED (p = 0.230). However, patients who were contacted directly indicated that they were more likely to adhere to received discharge instructions (p = 0.010). Neither did phoning patients significantly influence whether they followed-up with clinic providers (p = 0.999) or return to the same ED within 30 days (p = 0.999).

**CONCLUSIONS:**

Although there are often many complex factors influencing patients’ post-ED care decisions, the results from this smaller project indicated that contacting patients after ED discharge may help influence their perceptions of care and influence some follow-up care preferences.

## INTRODUCTION

Emergency Departments (ED) continue to face challenges in providing efficient, quality care in a climate of increased patient visits, longer waiting times, increased focus on hospital metrics, and a heightened emphasis on different aspects of patients’ perceptions of care.^1–4^ Hospital officials value patients’ overall satisfaction due to financial incentives (e.g., Medicare reimbursements) from reporting by entities such as *Press Ganey Associates*.^5^ As such, increased attention has been paid towards developing methods to improve patient perceptions of their care and future care preferences.^3,6,7^

The authors’ ED was similar to many departments in trying to identify cost-effective strategies to improve patient satisfaction in a climate where the volume of ED visits were at an all-time high.^8^ One proposed method was to contact patients by phone after ED discharge to inquire about their care perceptions and overall patient satisfaction. This prompted the authors to consider follow-up communication as an avenue of improvement at our healthcare system. However, the authors found that physicians were generally unable to contact patients after their ED visit for the purpose of obtaining patient follow-up communication.

Further, several studies have demonstrated a two-fold positive increase in perceptions of care received and likelihood to recommend a specific ED based on calling patients after ED discharge.^6,7,9^ There has also been renewed interest in other ways to attempt subsequent contact with former ED patients, including emails. For example, patients’ perceptions of ED care have been shown in one study to be higher when physicians later emailed patients.^10^ In addition, the results from two larger studies demonstrated an increased likelihood of patients to recommend an ED to friends and families after receiving a post-discharge phone contact.^11,12^

### Purpose of Study

The overall goal of this study was to investigate whether phoning patients who had completed a community-based ED visit influenced their later preferences concerning follow-up care and perception of our ED. The authors’ specific objectives were to identify whether contacting patients after ED discharge may influence their adherence with discharge instructions, whether they may be more likely to follow up with clinic-based providers, or if they were more likely to return to the same ED if changes in their conditions indicated. The authors also collected data concerning other factors previously shown to influence ED patient care perceptions such as wait time before receiving care, total hours of ED length of stay, etc.^13,14^

## METHODS

This study was a prospective descriptive study conducted between September 2017 through January 2018. Eligible patients had been evaluated and discharged from the authors’ community-based ED in the Detroit Michigan area, a Level II Trauma Center with 72,000+ annual visits. When possible, sample patients were phone contacted by the authors between 24 to 48 hours after ED discharge and either: a) spoken to directly, b) left a voicemail message, or c) recorded as never successfully contacted. In addition, paper surveys created by the authors with return self-addressed stamped envelopes were sent to each patients’ reported home address. Please see Figure 1 for an overall study flowchart detailing the ED visit to follow-up phone call to mailed survey process, and Figure 2 for a mailed paper survey template.

**Figure:**
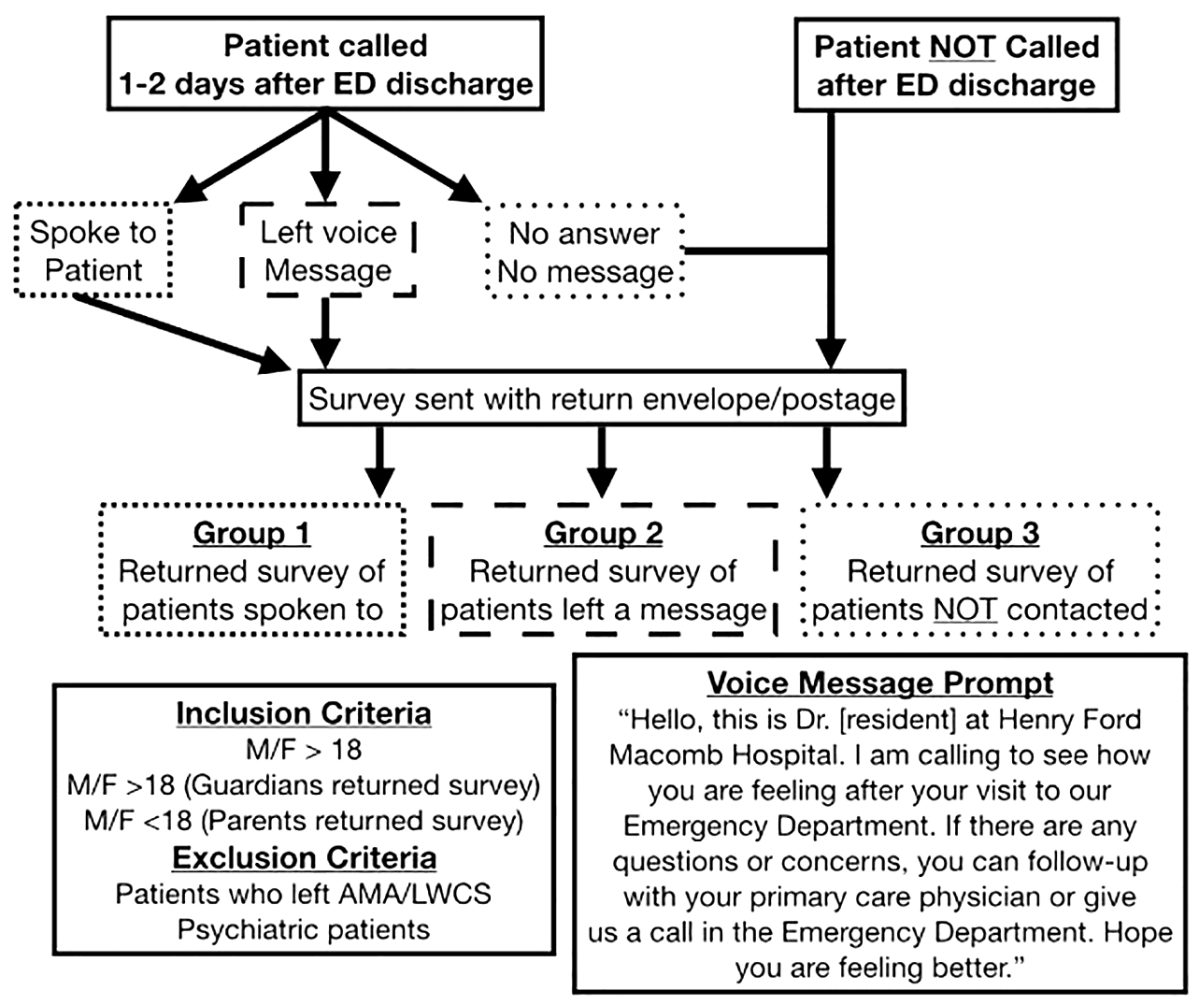
Figure 1 Study Flowchart, Inclusion and Exclusion Criteria, Voice Message Prompt

Patients with a documented history of a psychiatric illness, those who had left against medical advice (AMA) or left without completion of services were excluded from the study. If a patient was not directly contacted, a scripted voicemail message prompt was left to protect patient information. (Figure 1, bottom right) The authors institutional review board had approved the study prior to data collection.

### Study Outcomes

The first study outcome concerned whether patients were more or less likely to return to the same ED assessed on a five-point Likert-type scale survey. Patients who reported “5” were “very likely” to return to the ED versus “1” were “not very likely” to return for future evaluation. The authors hypothesized that patients who were phone contacted soon after ED discharge would be more satisfied with the overall care provided and more likely to return to the our ED again for future evaluations if needed.

Patients were also asked to report whether they had decided to: (a) adhere to ED discharge instructions and b) make a follow-up appointment with a clinic-based provider. During the study period, sample patients were also monitored in the electronic health record (EHR) for any returns to the same ED within 30 days for re-evaluation of the same chief complaint. Sample patients were monitored for return to the same ED to determine if speaking with patients after their ED discharge would help clarify their discharge instructions and manage their post-discharge expectations.

We had hypothesized that patients who were contacted on the phone would be more adherent to discharge recommendations. We also expected that patients who were spoken to after discharge would be more likely to follow-up with their clinic-based provider. We had also anticipated that patients who were directly spoken to would be less likely to return to the same ED for the same chief complaint within 30 days as contacted patients would be provided more guidance/reassurance to potentially circumvent their need to return to the ED.

### Data Analyses

Categorical data were summarized as counts and percentages. Survey responses “one” through “four” were grouped and compared to patients who reported “five” which is similar to how *Press Ganey* categorized survey responses.^5^ Between-group mean differences were first compared by calculating t-tests for independent measures (i.e., age, sex, number of past medical conditions, number of diagnosed conditions in ED, emergency severity index, wait time before receiving care, and length of stay). Categorical data were then compared using the chi-square test or Fisher’s exact test. For all analysis, a two-tailed p-value < 0.05 was observed as statistically significant.

Following the authors’ initial data entry using Microsoft Excel, Minitab 18 Statistical Software (State College, PA), or Langsrud online calculator^15^ were used by the campus-based analyst (see acknowledgements) to conduct selected analyses.

## RESULTS

There were a total of 96 (21.7%) surveys returned from the 443 sent to discharged ED sample patients. One response was excluded from analysis as no items were answered. Returned surveys were also divided into three sample subgroups: a) contacted over the phone (n = 65, 69.1% of total sample), b) left a voicemail message (n = 10, 10.6%), and c) never successfully contacted (n = 19, 20.2%). Prior to inferential analyses, independent variables (i.e., age, sex, number of past medical conditions, emergency severity index, wait time, length of stay (LOS) were compared among the three sample subgroups with no statistically significant differences found. (Table 1)

**Table:** Table 1 Patient Subgroup Characteristics

	**Spoke to Group (*n = 65*)**	**Message Group (*n = 10*)**	**No Contact Group (*n = 19*)**	***p*-value**
**Age**	63.4	46.1	63.9	0.96
**Male**	28	3	6	0.74
**Female**	39	7	13	
**Number of PMH**	6.4	2	5.5	0.13
**Diagnosed Conditions**	2	2	2.2	0.4
**Emergency Severity Index Score**	2.9	3.1	2.8	0.31
**Wait Time** **(in minutes)**	8.1	10.1	10.5	0.28
**LOS (in minutes)**	216.9	230.8	255.3	0.19

As shown in Table 2, patients who had been first phone contacted then self-reported via survey a somewhat higher likelihood of returning to the same ED as needed in the future compared to self-reported survey response patients who were never contacted, although not to a statistically significant degree (p = 0.060). Patients who were left a phone message after ED discharge were not significantly more likely to self-report intentions to return to the same ED again for future evaluations (p = 0.230). (Table 2)

**Table:** Table 2 Phone Contact Frequencies

**A**		
	**Spoke to Group (n = 64)**	**No Contact Group (n = 17)**
**Responded** **1 - 4**	29	12
**Responded 5**	35	5
		
**B**		
	**Message Group (n = 9)**	**No Contact Group (n = 17)**
**Responded** **1 - 4**	4	12
**Responded 5**	5	5

Chi-square analysis on likelihood of patients to return to ED for further evaluations. A) Patients who were spoken to trended to an increased likelihood of returning to the ED for future evaluations when compared to patients that were not contacted after ED discharge (p = 0.06). B) Patients who were left a message were not more likely to return to the ED for further evaluation when compared to patients that were not contacted after ED discharge (p = 0.23).

Additional outcome measures concerned whether phoning patients after discharge influenced their self-reported intent of following discharge instructions, self-reported intent to follow-up with a clinic-based provider, and the rate of return visits to the same ED. Patients who were phone contacted were significantly more likely to self-report an intention to follow discharge instructions compared to never-contacted patients (p = 0.010). However, speaking with patients did not significantly influence their self-reported intent of scheduling a follow-up with clinic providers (p = 0.099). Although a greater number of patients who were phone contacted returned to the same ED for the same chief complaint within a 30-day period (10 of 65, 15.4%) than non-contacted patients (3 of 19, 15.8%), this difference could not be inferentially examined due to inadequate frequency volume (i.e. small sub-sample) (p = 0.999).

## DISCUSSION

These results indicate that patients who were phone contacted after ED discharge tended to self-report a higher intent of adhering to discharge instructions, although not of returning to the same ED again for future evaluations. This finding matches a Cochrane meta-analysis examining post-discharge phone calls and patient expectations of care which did not find improved patient satisfaction from contacting patients.^16^ Neither did phone contacting sample patients after ED discharge result in an increased self-reported likelihood of intent to follow-up with a clinic-based provider.

Each sample patient spoken with on the phone self-reported following their ED discharge instructions. This finding was in agreement with studies showing increased discharge instruction adherence in patients discharged after hospital admission.^17^ We have since concluded that additional phone conversations concerning patients’ current medical conditions and what medications or further interventions might be needed if their symptoms changed.

These results failed to reveal a statistically significant reduction in return ED visits for the same chief complaint within a 30-day period. This finding is in agreement with previous studies showing that follow-up phone calls had not influenced 30-day hospital re-admission rates.^18^ However, this finding conflicted with a previous study showing a follow-up phone call reducing return visits within 30 days compared to non-contacted patients.^19^ Notably, four (6.3%) of the 64 patients who were successfully contacted were advised to return to the ED and ended up being admitted due to changes in their clinical conditions identified by interviewers. This finding suggests that speaking with some patients after ED discharge may provide an opportunity for risk mitigation as patients’ clinical conditions may change despite earlier ED care.

We acknowledge that our study results were likely affected by several design limitations. Some phone numbers used to contact patients that had been entered into the EHR may have been incorrect. These findings are based on patients’ self-reports about their adherence to discharge instructions and actual follow-up with clinic provider that could not be realistically confirmed. Although our sample size was smaller than some cited studies, our 21.7% survey response rate was similar to related studies.^10–12^ We should also acknowledge that some of our phone interview or survey questions may have been unclear to some patients.

## CONCLUSIONS

These results suggest that contacting certain higher-risk patients after their ED visit may be an effective strategy for helping influence follow-up care preferences and could improve perceptions of earlier ED care. Such interventions may also increase the likelihood of some patients to return to a familiar ED facility when needed. This can be an especially important step in coordinating patient care between ED and other clinic-based providers to help them avoid further health problems. A post-ED visit phone call may also convey to patients a level of concern and compassion that they may not have perceived during typically rushed ED encounters.

As ED physicians, we are tasked with treating a range of medical conditions while maintaining a high level of patient satisfaction. Strategies to influence care perceptions by former ED patients need to be further studied. Contacting patients after their ED discharge continues to be an intriguing potential strategy requiring further evaluation.

The authors declare no conflict of interest.

Submitted for publication May 2018.

Accepted for publication July 2018.

## ACKNOWLEDGMENTS

1. Robert Jarski, Ph.D., P.A. of Oakland University School of Health Sciences for providing statistical support and analyses;
2. Costandinos Tsagaratos, D.O. for taking an initial form of the survey and transcribing the revised study survey into the electronic health record for discharged patients.
3. Financial support for this project was also provided by a MSU Statewide Campus System 2017 Resident Research Support Grant.
4. The overall study findings were presented during an oral presentation at the Michigan College of Emergency Physicians Research Forum (April 2018).

### Figure 2 Mailed Patient Survey

[Patients Name] MRN: [Patients MRN]

Dear [Patients Name]

[Patient Address]

You have been automatically enrolled in a research project at Henry Ford Macomb Hospital. This study is optional but has the potential to greatly improve patient care at our facility. If you are willing to participate, please fill out the attached survey and return in the enclosed stamped envelope.

The protection of your personal information is very important to us. Specific patient information will be only handled by the private investigator. Your personal information is masked with a 4 digit number. All personal information will be destroyed after the survey is sent to you. No information on your conditions or why you were seen will be stored.

The information will not be shared with any other persons or institutions.

If you have any questions or concerns, please do not hesitate to call.

Sincerely,

Matthew C. Bombard, D.O.

Chief, Emergency Medicine Resident PGY IV

Michigan State University College of Osteopathic Medicine, Clinical faculty

HF MACOMB EMERGENCY

15855 19 Mile Road

Clinton Township MI 48038

[Emergency Department phone number]

ER Visit Date ___________ Survey ID __________

Patient Callback Survey

1. Were you examined and/or treated in the hallway or a room?

A. Hallway

B. Room

2. Did the Emergency Medicine Resident speak with you **AFTER** your Emergency Department visit?

A. **YES**, I spoke to the resident (**Go to Question 3**)

B. **NO**, I was left a message (**Go to Question 4**)

C. There was no phone call or voice message (**Go to Question 5**)

______________________________________________________________

3. **ANSWER ONLY IF YOU SPOKE WITH A RESIDENT**. Did that interaction AFTER your Emergency Department visit make you more or less likely to visit the Emergency Department again?

1 - Not very likely

2 - Not likely

3 - No opinion

4 - Likely

5 - Very likely

______________________________________________________________

4. **ANSWER ONLY IF A RESIDENT LEFT YOU A MESSAGE**. Did the voice message AFTER your Emergency Department visit make you more or less likely to visit the Emergency Department again?

1 - Not very likely

2 - Not likely

3 - No opinion

4 - Likely

5 - Very likely

______________________________________________________________

5. **ANSWER ONLY IF NO ONE CONTACTED YOU AFTER YOUR ER VISIT**. Would a follow up phone call after your Emergency Department visit make you more or less likely to visit the Emergency Department again?

1 - Not very likely

2 - Not likely

3 - No opinion

4 - Likely

5 - Very likely

______________________________________________________________

6. Did you continue the plan that was made in the Emergency Department?

YES

NO

7. Did you make an appointment with your Primary Care Physician (or a PCP to follow up with) after your Emergency Department Visit?

YES

NO

Thank you for completing this survey. Your response is important to providing a better experience for your next visit.
